# Changes of metabolic parameters, antioxidant capacity, and gut microbiota in response to substitution of ferrous sulfate with iron hydroxy methionine analog chelate in weaned piglets

**DOI:** 10.3389/fcimb.2025.1539607

**Published:** 2025-02-18

**Authors:** Yuemeng Fu, Guohui Zhou, Yuhang Liu, Xuejun Yuan, Ning Jiao, Wenbiao Lu, Weiren Yang

**Affiliations:** 1 Key Laboratory of Efficient Utilization of Non-Grain Feed Resources (Co-construction by Ministry and Province), Ministry of Agriculture and Rural Affairs, Shandong Provincial Key Laboratory of Animal Nutrition and Efficient Feeding, College of Animal Science and Technology, Shandong Agricultural University, Tai’an, China; 2 College of Life Sciences, Shandong Agricultural University, Tai’an, China; 3 Research and Development Department, Fujian Syno Biotech Co., Ltd., Fuzhou, China

**Keywords:** antioxidant, gut microbiota, metabolic parameters, organic iron, weaned piglets

## Abstract

**Introduction:**

Previous studies have suggested that dietary organic iron offers health advantages compared to its inorganic counterpart. However, the effects of iron hydroxy methionine analog chelate (Fe-HMA) supplementation in weaned piglets have not been fully explored. Therefore, this study aimed to investigate the effects of replacing ferrous sulfate with Fe-HMA as the iron source on serum biochemistry, antioxidant capacity, and gut microbiota in weaned piglets.

**Methods:**

One hundred and twenty weaned piglets were randomly allocated to two treatment groups. Each group contained four replicates, with 15 pigs per replicate. Piglets were fed either 100 mg Fe/kg in the form of ferrous sulfate (Fe-sulfate group) or 50 mg Fe/kg in the form of Fe-HMA (Fe-HMA group) as the iron source for 28 days.

**Results and discussion:**

Results showed that supplementing Fe-HMA as an iron source significantly increased the levels of triglycerides and glucose in portal venous serum, albumin in both serum and portal venous serum and decreased serum low-density lipoprotein level in weaned piglets. Additionally, Fe-HMA supplementation significantly reduced serum and liver malondialdehyde levels, while increasing catalase (CAT), glutathione peroxidase (GSH-Px), total superoxide dismutase, and manganese superoxide dismutase levels in serum, as well as GSH-Px and CAT levels in the liver. Moreover, Fe-HMA regulated the intestinal microbiota composition, notably increasing the relative abundance of Proteobacteria and decreasing microbes involved in aromatic_compound_degradation. In conclusion, dietary replacing inorganic iron with Fe-HMA improved metabolic parameters and antioxidant capacity, and regulated gut microbiota composition in weaned piglets.

## 1 Introduction

Iron, an essential trace element, plays a vital role in piglet growth by supporting various physiological processes, including oxygen transport, DNA synthesis, antioxidant enzyme formation, and energy metabolism (Sun et al., 2022). Iron deficiency can lead to vigor reduction, appetite loss, irritability, and pica, which in turn can result in malnutrition and ultimately affect growth (Pasricha et al., 2021). However, the iron reserves in weaned piglets are insufficient to support rapid growth, which can make them susceptible to iron deficiency (Heidbüchel et al., 2019; Ding et al., 2021). In addition, weaning represents a critical phase in piglet production, marked by separation from the mother and the transition to solid feed (Tang et al., 2022). These changes exert stress on piglets, thereby affecting their antioxidant status and nutrient absorption, and potentially hindering growth as their intestine is under development (Cao et al., 2018). Moreover, excessive iron supplementation can exacerbate weaning stress (Ding et al., 2020b; Ding et al., 2021). Therefore, maintaining an appropriate iron level and ensuring adequate iron absorption in weaned piglets is an effective strategy to support their health (Kim et al., 2018).

The National Research Council (NRC, 2012) recommends that the dietary iron requirement is 100 mg/kg in weaned piglets. However, excess iron is often added to diets to ensure sufficient iron intake in practical production (Kim et al., 2018). The iron sources currently can be broadly classified into inorganic and organic forms. The ions in inorganic iron additives, such as ferrous sulfate, are unstable and prone to redox reactions, which can lead to toxicity and oxidative stress (Galaris et al., 2019). Additionally, the low bioavailability of inorganic iron, coupled with the underdeveloped intestines of weaned piglets, can lead to substantial iron excretion with feces, then resulting in iron waste and environmental contamination (Zeng et al., 2023). Organic iron is typically considered to be more efficiently absorbed than inorganic iron (Feng et al., 2019). Studies have shown that organic iron, even at lower doses or half the dose of inorganic iron, has no adverse effects and may even offer benefits in weaned piglets (Feng et al., 2007; Feng et al., 2009). Normally, only ferrous ions can be absorbed by intestine (Shubham et al., 2020). The types of organic iron include simple organic acid iron salts, such as ferrous citrate and ferrous fumarate, as well as chelated iron complexes, in which the iron is bound to amino acids or other organic substances (Ma et al., 2022). Among these, amino acid-chelated iron is especially noteworthy due to its greater stability compared to organic acid iron salts (Predieri et al., 2009). Hydroxy methionine analogs (HMA) can form two chelate rings with metal ions to enhance their stability and absorption (Biagi et al., 2004). It has been reported that Fe-HMA did not cause toxicity in CACO-2 cells, which highlighted its promise as an effective and safe iron supplement (Predieri et al., 2003). The previous study showed that replacing inorganic trace elements with HMA chelate trace elements enhanced serum antioxidant capacity and improved immune function in finishing pigs (Chen et al., 2022). Additionally, a study on juvenile grouper demonstrated that bioavailability of Fe-HMA was approximately 1.3 times greater than that of ferrous sulfate (Huang et al., 2018). Organic iron can also influence intestinal microbiota composition. For instance, dietary supplementation with 84 mg/kg yeast iron increased beneficial bacteria like Firmicutes, Blautia, and Peptococcus levels in the cecum of weaned piglets compared with dietary 104 mg/kg inorganic iron (Zeng et al., 2023). However, the specific effects of Fe-HMA on weaned piglets health have yet to be fully elucidated.

Therefore, this study aims to investigate the effects of replacing ferrous sulfate with Fe-HMA on metabolic parameters, antioxidant capacity, and gut microbiota in weaned piglets, which could provide the basis for the application of Fe-HMA in weaned piglets.

## 2 Materials and methods

### 2.1 Animal care

The animal experiment was approved by the Animal Care and Use Committee of Shandong Agricultural University (protocol code SDAUA-2023-327).

### 2.2 Piglets and management

A total of 120 healthy 35 d-old weaned piglets (Duroc × Landrace × Large White) with an average body weight of 11.09 ± 0.164 kg were used in a 28-d trial. All piglets were randomly allocated into two groups, each with 4 replicates of 15 pigs per pen in the same room. All pigs were fed a basal diet (**Table 1**) which was formulated with reference to the National Research Council (2012) to meet or exceed nutritional requirements, except for iron. In the Fe-sulfate group, the diet was supplemented with 100 mg Fe/kg in the form of ferrous sulfate monohydrate, while the Fe-HMA group received 50 mg Fe/kg supplementation in the form of Fe-HMA (Changsha Xinjia Bio-Engineeriong Co., Ltd., Changsha, China). The supplementation amount was based on the iron content. A three-day acclimation period was provided to help piglets adjust to the living environment before the official trial. The diets were gradually mixed with the experimental diet to ensure a smooth transition to the experimental diet. Throughout the experiment, piglets were housed in a temperature-controlled room (25-28°C) with *ad libitum* access to feed and water.

### 2.3 Sample collection and preparations

At 28 day of the experiment, one piglet from each replicate was selected to collect 10 mL blood and portal venous blood from the jugular vein and dissected thoracic cavity respectively. Serum was obtained from blood samples after centrifugation at 3500 rpm for 15 minutes and was immediately stored at –20°C until analysis.

Approximately 2 g of liver tissue were collected in 2 mL frozen storage tubes, immediately immersed in liquid nitrogen, and then stored at –80°C until antioxidant determination. Cecal chyme were collected into 5 mL sterilized fecal containers for microbiota composition analysis.

### 2.4 Serum biochemistry

Glucose (GLU), total protein (TP), albumin (ALB), urea nitrogen (UREA), triglycerides (TG), total cholesterol (TC), high-density lipoproteins (HDL) and low-density lipoproteins (LDL) in serum and portal venous serum were analyzed on COBUS MIRA Plus fully automated biochemical analyzer (Roche, Indianapolis, USA).

### 2.5 Malondialdehyde and antioxidant indicators

MDA and antioxidants [copper-zinc superoxide dismutase (CuZn-SOD), superoxide dismutase (SOD), Mn superoxide dismutase (Mn-SOD), glutathione peroxidase (GSH-Px), and catalase (CAT)] in serum, portal venous serum and liver were determined using commercial kits from Nanjing Jiancheng Bioengineering Institute (Jiangsu, China) following manufacturer instructions according to previous studies (Sun et al., 2023). Briefly, serum and portal serum were analyzed directly. Liver tissues were homogenized with saline (w/v, 1:9) to obtain supernatant, which was subsequently used for antioxidant indicators determination. The MDA content and antioxidant enzyme activities in liver were normalized to the total protein concentration (per mg protein) of each sample, which was measured using a commercial protein assay kit (Nanjing Jiancheng Bioengineering Institute, Jiangsu, China).

### 2.6 Microbial 16S rRNA gene sequencing and analysis of cecal content

Total bacterial genomic DNA were extracted from the cecal chyme with the E.Z.N.A.TM stool DNA kit (Omega Bio-Tek, GA, USA). The spectrophotometer and 1% agarose gels electrophoresis were used for monitoring DNA concentration and purity followed by diluting the extracted DNA to 1 ng/mL. The primers 27F (5′-AGRGTTYGATYMTGGCTCAG-3′) and 1492R (5′-RGYTACCTTGTTACGACTT-3′) were adopted to amplify V1-V9 region of the bacteria 16S rRNA gene (Dong et al., 2024; Li et al., 2024). Amplicons were extracted by 2% agarose gels electrophoresis, purified with AxyPrep DNA Gel Extraction Kit (Axygen Biosciences, CA, USA) and quantified with QuantiFluor^®^ dsDNA System (Promega Biotech, WI, USA). Subsequently, SMRTbell libraries were constructed for sequencing on `PacBio Sequel cells. Amplicon sequencing was performed by Shanghai Majorbio Bio-Pharm Technology Co., Ltd. (Shanghai, China). The Uparse pipeline (v.11) was used to cluster high-quality sequences with 97% similarity into identical operational taxonomic units (OTU). Alpha diversity including ACE, Chao 1, Shannon and Simpson indices was revealed by rarefaction analysis using Mothur (v.1.30.2) (Yang et al., 2020). Beta diversity of hierarchical clustering and principal coordinate analysis (PCoA) based on Bray–Curtis dissimilarity matrices were performed by Quantitative Insights Into Microbial Ecology (QIIME v.1.9.1) (Li et al., 2024). Wilcoxon rank-sum test and Linear discriminant analysis (LDA) combined effect size measurements (LEfSe) were employed to identify bacterial differences between groups. The identification of bacterial differences between groups was accomplished using both the Wilcoxon rank-sum test and Linear Discriminant Analysis Effect Size (LEfSe). Microbial community functional prediction was conducted using Functional Annotation of Prokaryotic Taxa (FAPROTAX) (Li et al., 2020).

### 2.7 Statistical analysis

All the indices were determined using individual weaned piglets as the experimental unit. Data normality was assessed using the Shapiro-Wilk test (W > 0.05). All experimental data were analyzed using an independent t-test in SAS 9.6, with significance defined at *P* < 0.05.

## 3 Results

### 3.1 Serum biochemistry

As shown in **Table 2**, serum ALB was significantly increased while LDL was significantly reduced in Fe-HMA group compared with Fe-sulfate group (*P* < 0.05). No significant differences were observed in TP, UREA, GLU, TG, TCHO, and HDL between the two groups (*P* > 0.05).

**Table 1:** Ingredient composition and nutritional values of basal diets (air-dried basis).

Items	Content
Ingredients	
Corn	59.15
Soybean meal	16.0
Expanded soybean	13.0
Fish meal	2.80
Whey	3.00
Soybean oil	2.00
Limestone	0.80
Calcium hydrogen phosphate	1.00
NaCl	0.25
Premix^1^	2.00
Total	100.0
Nutrient^2^	
Digestible energy, MJ/kg	14.85
Crude protein, %	18.88
Standard total gastrointestinal digestibility phosphorus, %	0.36
Calcium, %	0.73
Lysine, %	1.23
Threonine, %	0.74
Methionine, %	0.47
Tryptophan, %	0.20
Methionine + Cysteine, %	0.69
Fe, mg/kg	65.88

^1^Premix is provided per kg of the diet: vitamin A, 1750 IU; vitamin D_3_, 200 IU; vitamin E, 11 IU; vitamin K_3_, 0.5 mg; vitamin B_1_, 1 mg; vitamin B_2_, 3 mg; vitamin B_12_, 15 μg; biotin, 0.05 mg; folic acid, 0.3 mg; pantothenic acid, 9 mg; pyridoxine, 3.0 mg; Mn, 50 mg; Zn, 95 mg; Cu, 125 mg; I, 0.14 mg; Se, 0.25 mg.

^2^Nutrient levels are calculated. Iron level was determined experimentally.

**Table 2:** Effects of replacing inorganic iron with hydroxy methionine analog chelated iron (Fe-HMA) on serum biochemistry of piglets.

Items	Fe-sulfate^1^	Fe-HMA^2^	SEM	*P* value
TP, g/L	55.95	57.30	0.861	0.476
ALB, mmol/L	17.75	20.93	0.744	0.015
UREA, mmol/L	3.37	3.44	0.061	0.627
GLU, mmol/L	7.87	8.99	0.364	0.127
TG, mmol/L	0.83	0.82	0.059	0.925
TCHO, mmol/L	2.90	2.78	0.056	0.338
HDL, mmol/L	0.77	0.78	0.011	0.683
LDL, mmol/L	1.50	1.41	0.023	0.039

TP, total protein; ALB, albumin; UREA, urea nitrogen; GLU, glucose; TG, triglyceride; TCHO, total cholesterol; HDL, high-density lipoprotein; LDL, low-density lipoprotein; SEM, standard error of the means.

^1^Basal diet supplementing 100 mg Fe/kg in the form of ferrous sulfate.

^2^Basal diet supplementing 50 mg Fe/kg in the form of Fe-HMA.

### 3.2 Portal venous serum biochemistry

As shown in **Table 3**, dietary Fe-HMA as the iron source significantly elevated ALB, GLU, TG and HDL levels in portal venous serum compared with Fe-sulfate group (*P* < 0.05). Meanwhile, compared with Fe-sulfate group, portal venous serum UREA and TCHO showed a tendency increase in Fe-HMA (0.05 < *P* < 0.10). There were no significant differences in TP and LDL between the two groups (*P* > 0.05).

**Table 3:** Effects of replacing inorganic iron with Fe-HMA on portal venous serum biochemistry of piglets.

Items	Fe-sulfate^1^	Fe-HMA^2^	SEM	*P* value
TP, g/L	49.90	56.25	2.011	0.117
ALB, mmol/L	14.70	20.20	1.246	< 0.001
UREA, mmol/L	3.91	4.43	0.156	0.086
GLU, mmol/L	12.62	16.83	0.975	0.002
TG, mmol/L	0.61	0.94	0.075	0.001
TCHO, mmol/L	2.44	2.66	0.064	0.060
HDL, mmol/L	0.57	0.82	0.058	0.004
LDL, mmol/L	1.39	1.37	0.012	0.448

TP, total protein; ALB, albumin; UREA, urea nitrogen; GLU, glucose; TG, triglyceride; TCHO, total cholesterol; HDL, high-density lipoprotein; LDL, low-density lipoprotein; SEM, standard error of the means.

^1^Basal diet supplementing 100 mg Fe/kg in the form of ferrous sulfate.

^2^Basal diet supplementing 50 mg Fe/kg in the form of Fe-HMA.

### 3.3 MDA content and antioxidant enzyme activity in serum

The MDA content and antioxidant enzyme activity in serum are displayed in **Table 4**. Fe-HMA significantly reduced MDA content while increasing GSH-Px, CAT, T-SOD and Mn-SOD activities in serum (*P* < 0.05). No significant difference was observed in CuZn-SOD activity between the two groups (*P* > 0.05).

**Table 4:** Effects of replacing inorganic iron with Fe-HMA on serum antioxidants of piglets.

Items	Fe-sulfate^1^	Fe-HMA^2^	SEM	*P* value
MDA, nmol/mL	5.92	3.37	0.600	0.004
GSH-Px, U/mL	604.10	662.69	15.359	0.044
CAT, U/mL	104.97	114.12	2.170	0.018
T-SOD, U/mL	181.83	208.94	6.579	0.023
CuZn-SOD, U/mL	121.79	127.21	1.805	0.141
Mn-SOD, U/mL	60.04	81.73	5.758	0.048

MDA, malonaldehyde; GSH-Px, glutathione peroxidase; CAT, catalase; T-SOD, total superoxide dismutase; CuZn-SOD, copper and zinc superoxide dismutase; Mn-SOD, manganese superoxide dismutase; SEM, standard error of the means.

^1^Basal diet supplementing 100 mg Fe/kg in the form of ferrous sulfate.

^2^Basal diet supplementing 50 mg Fe/kg in the form of Fe-HMA.

### 3.4 MDA content and antioxidant enzyme activity in portal venous serum

As shown in **Table 5**, a decreased tendency was observed in MDA content in Fe-HMA group (0.05 **<** *P* < 0.10). There were no significant differences between the two groups in GSH-Px, CAT, T-SOD, Mn-SOD and CuZn-SOD activities (*P* > 0.05).

**Table 5:** Effects of replacing inorganic iron with Fe-HMA on portal venous serum antioxidants of piglets.

Items	Fe-sulfate^1^	Fe-HMA^2^	SEM	*P* value
MDA, nmol/mL	6.14	4.96	0.343	0.082
GSH-Px, U/mL	201.55	211.59	5.833	0.432
CAT, U/mL	76.10	77.60	2.324	0.773
T-SOD, U/mL	166.51	175.54	5.697	0.491
CuZn-SOD, U/mL	120.28	127.54	8.772	0.725
Mn-SOD, U/mL	46.23	48.00	3.946	0.850

MDA, malonaldehyde; GSH-Px, glutathione peroxidase; CAT, catalase; T-SOD, total superoxide dismutase; CuZn-SOD, copper and zinc superoxide dismutase; Mn-SOD, manganese superoxide dismutase; SEM, standard error of the means.

^1^Basal diet supplementing 100 mg Fe/kg in the form of ferrous sulfate.

^2^Basal diet supplementing 50 mg Fe/kg in the form of Fe-HMA.

### 3.5 MDA content and antioxidant enzyme activity in liver

As shown in **Table 6**, MDA content in liver was significantly decreased in Fe-HMA group (*P* < 0.05). Meanwhile, Fe-HMA significantly increased GSH-Px and CAT activities in the liver (*P* < 0.05). No significant differences were found in T-SOD, CuZn-SOD and Mn-SOD activities in liver between the two groups (*P* > 0.05).

**Table 6:** Effects of replacing inorganic iron with Fe-HMA on liver antioxidants of piglets.

Items	Fe-sulfate^1^	Fe-HMA^2^	SEM	*P* value
MDA, nmol/mgprot	5.53	3.80	0.465	0.040
GSH-Px, U/mgprot	100.25	116.21	3.881	0.024
CAT, U/mgprot	106.07	119.02	3.379	0.041
T-SOD, U/mgprot	27.47	28.95	0.613	0.257
CuZn-SOD, U/mgprot	21.01	20.72	0.220	0.556
Mn-SOD, U/mgprot	6.48	8.23	0.567	0.162

MDA, malonaldehyde; GSH-Px, glutathione peroxidase; CAT, catalase; T-SOD, total superoxide dismutase; CuZn-SOD, copper and zinc superoxide dismutase; Mn-SOD, manganese superoxide dismutase; SEM, standard error of the means.

^1^Basal diet supplementing 100 mg Fe/kg in the form of ferrous sulfate.

^2^Basal diet supplementing 50 mg Fe/kg in the form of Fe-HMA.

### 3.6 Cecal bacteria community

#### 3.6.1 Diversity

**Figure 1:**
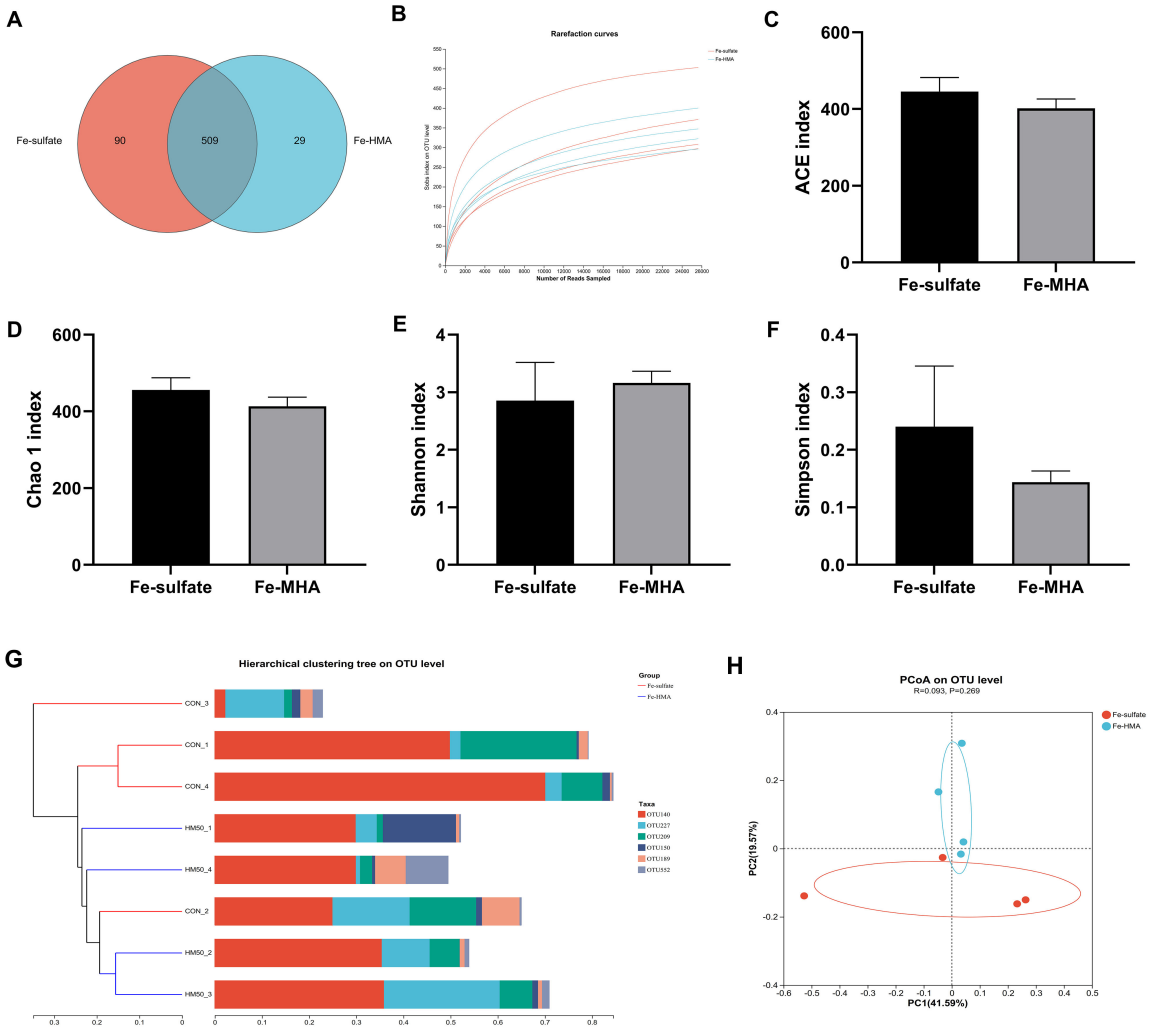
Effects of replacing inorganic iron with hydroxy methionine analog chelated iron (Fe-HMA) on cecal microbial diversity and composition of piglets. **(A)** Venn diagram; **(B)** Rarefaction curves; **(C)** ACE index; **(D)** Chao 1 index; **(E)** Shannon index; **(F)** Simpson index; **(G)** Hierarchical clustering tree on OTU level; **(H)** Principal coordinate analysis (PCoA) based on Bray–Curtis distances. Fe-sulfate, piglets fed the basal diet with 100 mg Fe/kg in the form of ferrous sulfate monohydrate; Fe-HMA, piglets fed the basal diet with 50 mg Fe/kg in the form of Fe-HMA. Values are depicted as means ± SEM (standard error).

The Venn diagram showed that a total of 628 OTUs were identified in the cecal contents of weaned piglets (**Figure 1A**). There were 509 equivalent OTUs between the two treatments, whereas 90 unique OTUs were in Fe-sulfate and 29 in Fe-MHA, respectively. Rarefaction curves of pooled samples were provided at an OTU definition of 97% identity and it tended to reach plateau with the number of reads sampled increased indicating an adequate sequence number (**Figure 1B**). No significance was observed between the two groups in alpha diversity indices of ACE index (**Figure 1C**), Chao 1 index (**Figure 1D**), Shannon index (**Figure 1E**) and Simpson index (**Figure 1F**) (*P* > 0.05). In the beta diversity analysis, hierarchical clustering and PCoA analysis based on bray_curtis distance (**Figure 1G**) were used to measure the similarity and differences of bacterial community composition in cecal contents between the two treatments. Hierarchical clustering tree at OUT level exhibited that the differences of cecal microbial flora in Fe-HMA group are smaller, which was also revealed by PCoA plot (**Figure 1H**) with the obviously separated samples between the two groups.

#### 3.6.2 Species composition

**Figure 2:**
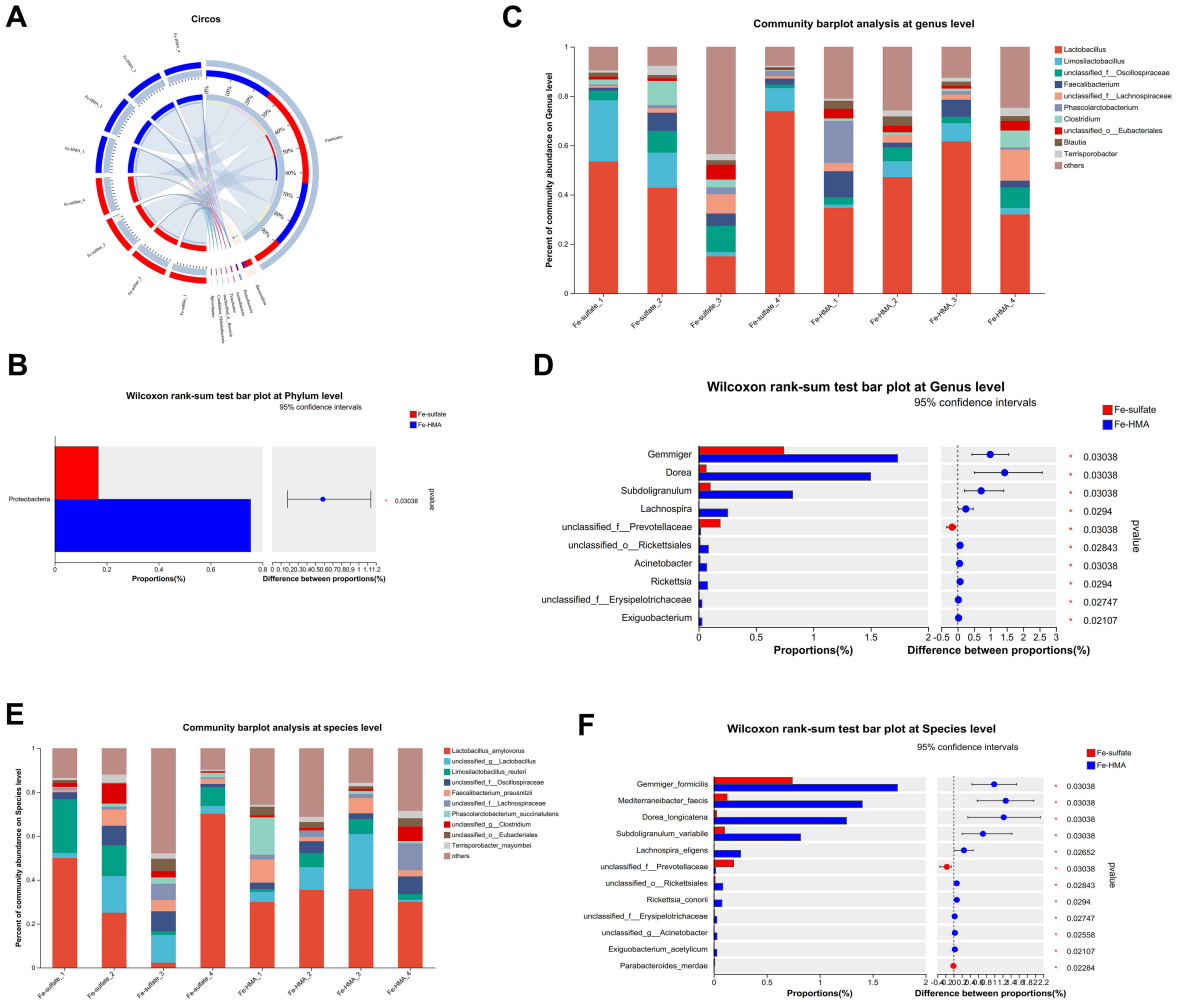
Effects of replacing inorganic iron with Fe-HMA on cecal microbial community at phylum, genus and species level. **(A)** Circos diagram at phylum level; **(B)** Wilcoxon rank-sum test bar plot at phylum level. **(C)** Community barplot analysis at genus level; **(D)** Wilcoxon rank-sum test bar plot at genus level; **(E)** Community barplot analysis at species level; **(F)** Wilcoxon rank-sum test bar plot at species level. Fe-sulfate, piglets fed the basal diet with 100 mg Fe/kg in the form of ferrous sulfate monohydrate; Fe-HMA, piglets fed the basal diet with 50 mg Fe/kg in the form of Fe-HMA.

The circos plot at phylum level showed that the primary dominant phylum of all samples was Firmicutes while the secondary dominant phylum was Bacteroidetes (**Figure 2A**). However, the Proteobacteria proportions were significantly elevated by Fe-HMA supplementation compared to Fe-sulfate (**Figure 2B**). The bacterial community bar plots of top 10 genera and species are shown in **Figure 2C** (at genus level) and **Figure 2E** (at species level). As shown in **Figure 2C**, *Lactobacillus* was the most prevalent genus of all samples. The proportion of *Gemmiger*, *Dorea*, *Subdoligranulum*, *Lachnospira*, *unclassified_o_Rickettsiales*, *Acinetobacter*, *Rickettsia*, *unclassified_f_Erysipelotrichaceae* and *Exiguobacterium* were significantly increased in Fe-HMA group while *unclassified_f_Prevotellaceae* was significantly decreased (*P* < 0.05) (**Figure 2D**). The primary dominant species was *Lactobacillus_amylovorus* in all samples (**Figure 2E**). Fe-HMA supplementation significantly increased the richness of *Gemmiger_formicilis*, *Mediterraneibacter_faecis*, *Dorea_longicatena*, *Subdoligranulum_variabile*, *Lachnospira_eligens*, *unclassified_o:Rickettsiales*, *Rickettsia_conorii*, *unclassified_f:Erysipelotrichaceae*, *unclassified_g:Acinetobacter and Exiguobacterium_acetylicum* and decreased *unclassified_f:Prevotellaceae* and *Parabacteroides_merdae* (*P* < 0.05) (**Figure 2F**).

**Figure 3:**
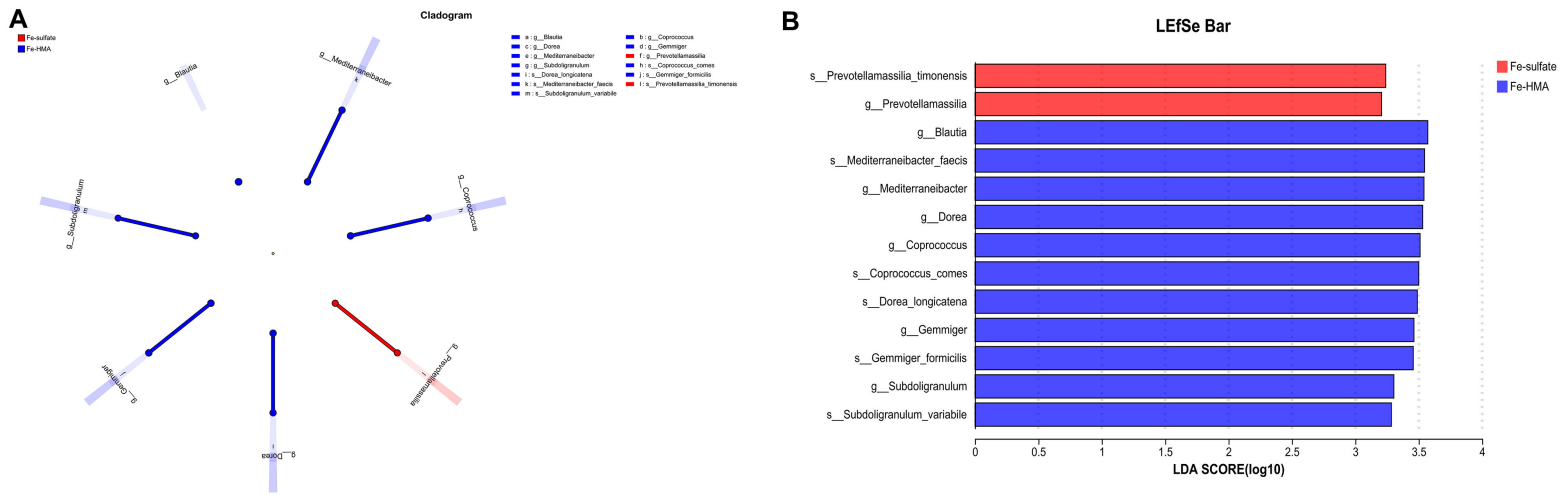
Effects of replacing inorganic iron with Fe-HMA on LEfSe analysis of piglets. **(A)** LEfSe multilevel taxonomic hierarchical tree diagram; **(B)** Histogram of LDA scores of microbiota with a threshold value of 4, showing features with differential abundance between groups. Fe-sulfate, piglets fed the basal diet with 100 mg Fe/kg in the form of ferrous sulfate monohydrate; Fe-HMA, piglets fed the basal diet with 50 mg Fe/kg in the form of Fe-HMA.

To further explore the most discriminant biomarker in the cecal microbiota between the two groups, LEfSe analysis was conducted (**Figure 3**). The results showed that *Prevotellamassilia* enriched in Fe-sulfate group, and *Blautia*, *Mediterraneibacter*, *Dorea*, *Coprococcus*, *Gemmiger* and *Subdoligranulum* were more abundant in Fe-HMA group at genus level. Meanwhile, *Prevotellamassilia_timonensis* was enriched in Fe-sulfate group, and *Mediterraneibacter_faecis*, *Coprococcus_comes*, *Dorea_longicatena*, *Gemmiger_formicilis* and *Subdoligranulum_variabile* were more abundant in Fe-HMA group at species level.

#### 3.6.3 Functional prediction

**Figure 4:**
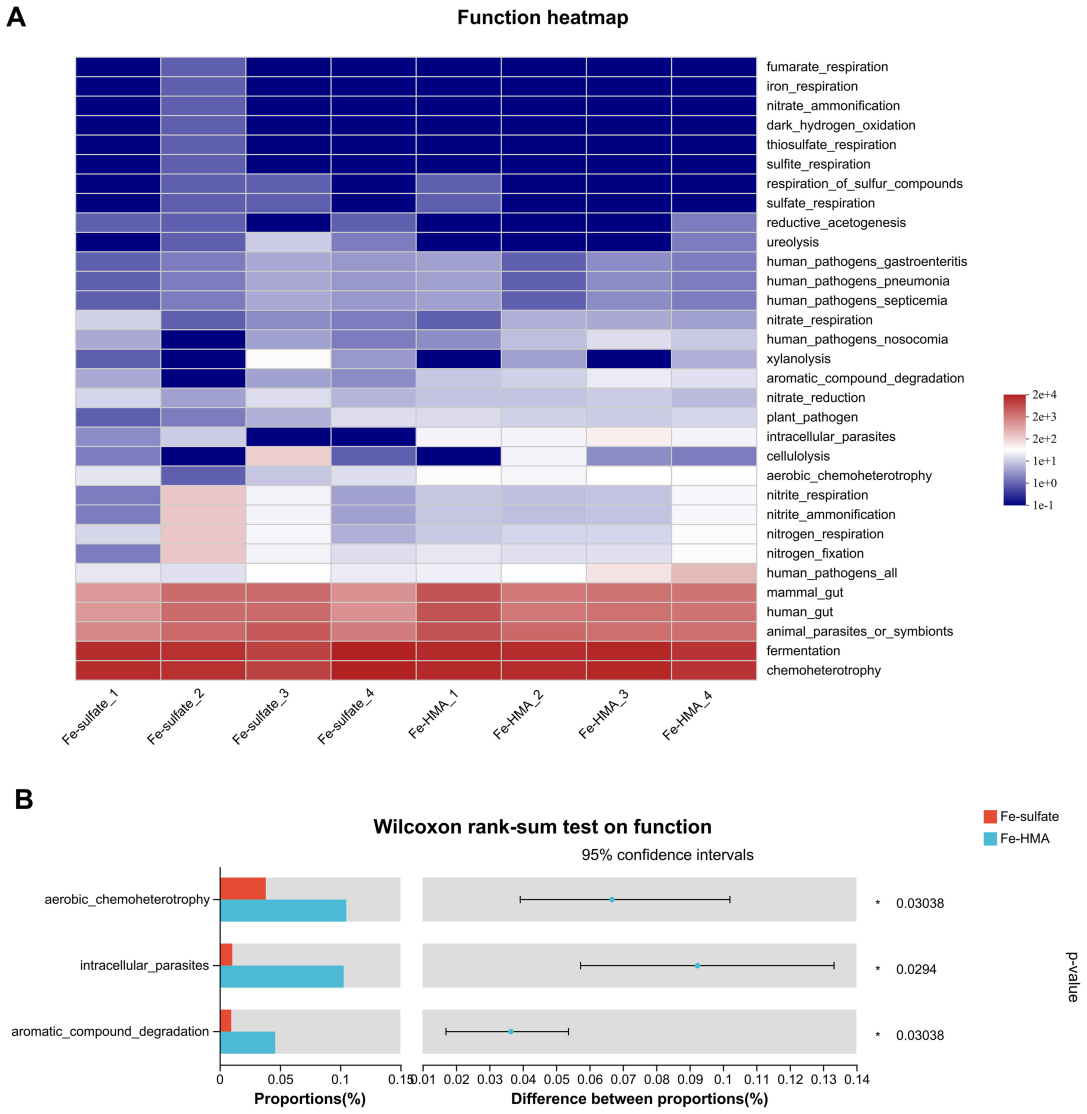
Effects of replacing inorganic iron with Fe-HMA on bacterial relative abundance of predicted function. **(A)** Heatmap of top 32 predicted microbial functions; **(B)** Wilcoxon rank-sum test bar plot on predicted function of gut bacteria. Fe-sulfate, piglets fed the basal diet with 100 mg Fe/kg in the form of ferrous sulfate monohydrate; Fe-HMA, piglets fed the basal diet with 50 mg Fe/kg in the form of Fe-HMA.

The heatmap of predicted microbial function by FAPROTAX is shown in **Figure 4A**. Around 32 enriched functions were identified between the two groups in weaned piglets. Among these, three functions (aerobic_chemoheterotrophy, intracellular_parasites, and aromatic_compound_degradation) were significantly enriched in the Fe-HMA group (*P* < 0.05) (**Figure 4B**)

## 4 Discussion

Serum biochemistry can reflect metabolic status and liver health in animals. ALB, a liver-secreted protein that maintains osmolality and transports nutrients, serves as the indicator of the liver’s protein synthesis capacity (Rothschild et al., 1988; Regmi et al., 2018). Iron plays a critical role in ATP synthesis, which provides the necessary energy for ALB secretion (Rothschild et al., 1988). A previous study demonstrated that dietary supplementation with iron chelates with lysine and glutamic acid at 400 mg/kg and 800 mg/kg significantly increased serum ALB levels in broilers (Han et al., 2022). In the present study, elevated ALB levels in both serum and portal serum of weaned piglets suggest that Fe-HMA may enhance liver function by providing highly bioavailable iron, compared to the inorganic iron group (Regmi et al., 2018). However, the content of LDL is associated with atherosclerotic diseases (Penson et al., 2020). In the present study, Fe-HMA reduced serum LDL level in weaned piglets, contributing to maintaining organism health (Penson et al., 2020). The hepatic portal vein, a major vein that enters the liver at the hilum hepatis after merging with the mesenteric veins from the digestive tract, plays a critical role in nutrient absorption and hormone metabolism (Han et al., 2021; He et al., 2023). In this study, the levels of TG and GLU in portal vein serum were elevated in the Fe-HMA group, suggesting Fe-HMA supplementation increased intestinal absorption of nutrients. Overall, Fe-HMA improved the serum biochemical profile of weaned piglets, contributing to improved health.

The weaning of pigs is usually accompanied by oxidative stress, which can be influenced by iron (Ma et al., 2014; Ding et al., 2021). A study showed that hydroxy methionine analogs could improve the antioxidant capacity of organisms (Kuang et al., 2012). MDA, a key indicator of oxidative stress, was significantly reduced in both serum and liver in the Fe-HMA group, indicating a decrease in lipid peroxidation levels in piglets (Zheng et al., 2013; Sun et al., 2023). However, CAT, SOD and GSH-Px are essential enzymes in animal antioxidant system (Feng et al., 2009; Zeng et al., 2023). Studies have reported organic iron can significantly enhance the antioxidant capacity of animals. For instance, supplementing yeast iron as the iron source in weaned piglets’ diets increased activities of CAT, GSH-Px, SOD and T-AOC in serum compared to inorganic iron (Zeng et al., 2023). Meanwhile, organic iron source, even at a lower dosage than inorganic iron, can improve antioxidant capacity. A study showed 90 mg/kg of glycine-chelated iron significantly elevated hepatic SOD and SDH relative mRNA expressions in weaned piglets compared to the 120 mg/kg ferrous sulfate addition (Feng et al., 2009). In this study, Fe-HMA increased serum activities of CAT, GSH-Px, T-SOD and Mn-SOD, as well as hepatic GSH-Px and CAT, indicating that Fe-HMA can enhance antioxidant capacity.

The bacteria in the intestine act as a barrier against pathogens, and their changes are closely linked to the health of piglets. Similar to dietary addition of iron made by saccharomyces cerevisiae (Zeng et al., 2023), dietary Fe-HMA supplementation had no significant effects on alpha indices including Simpson, Shannon and Chao1 compared with ferrous sulfate in the present study. The predominant bacteria at phylum level in the piglet cecum is Firmicutes (Chen et al., 2020). In this study, Firmicutes constituted the largest proportion of the gut microbiota in weaned piglets, indicating that the type of iron source had little effect on the prevalence of this dominant phylum. However, the iron source influenced the composition of other microbial communities within the gut. The Wilcoxon rank-sum test showed that the differential microorganism in the gut of piglets supplemented with the two iron sources at the phylum level was Proteobacteria, a potential pathogenic bacteria containing the most genera of bacteria that catalyze the oxidation of iron (Hedrich et al., 2011). The previous study showed that the abundance of Proteobacteria in cecal increased in weaned piglets fed with diet supplemented with a high level of 3000 mg/kg FeSO_4_ (Ding et al., 2020a). The organic iron supplementation has been shown to increase Proteobacteria relative abundance in intestine. A previous study showed that oral iron administration 25 mg/d Fe (ferrous glycine) improved the abundance of Proteobacteria in ileum, cecum and colon in the fourth day of newborn piglets (Dong et al., 2023). Zeng et al. (2023) reported that dietary supplemented 104 mg/kg and 124 mg/kg iron made by Saccharomyces cerevisiae increased the abundance of Proteobacteria in cecum. Similarly, the addition of Fe-HMA to the diet elevated the relative abundance of Proteobacteria in cecum of weaned piglets in the present study. *Gemmiger*, *Dorea*, and *Subdoligranulum* are the butyrate-producing genera (Lu et al., 2023; Ma et al., 2024; Visuthranukul et al., 2024). Butyrate is known for its anti-inflammatory properties and its ability to ameliorate intestinal inflammatory diseases (Zhang et al., 2021). A previous study showed that oral administration of *Escherichia coli* significantly reduced the abundance of *Gemmiger* in the colon of weaned piglets (Wang et al., 2024). Similarly, the abundance of *Gemmiger_formicilis* and *Subdoligranulum* was reported to decrease in the intestines of patients with inflammatory bowel disease (Ning et al., 2023; Visuthranukul et al., 2024). However, Fe-HMA supplementation increased the abundance of *Gemmiger*, *Dorea*, and *Subdoligranulum* in this study, suggesting its potential role in alleviating intestinal inflammation. Uremic toxins, including indolyl sulfate, p-Cresol sulfate and phenylacetylglutamine, can be produced by the metabolism of aromatic amino acids (Vacca et al., 2020). However, the proportion of microorganisms with aromatic_compound_degradation function increased in Fe-HMA group in this study, which may lead to lower toxins produced by aromatic compounds. Overall, Fe-HMA supplementation altered the intestinal bacterial composition in weaned piglets, which may impact intestinal health. However, their specific implications in intestine required further investigation.

## 5 Conclusions

In conclusion, Fe-HMA supplementation improved metabolic parameters, enhanced antioxidant capacity, and modulated gut microbiota composition in weaned piglets compared with Fe-sulphate. These improvements also supported liver function and intestinal nutrient absorption, potentially contributing to overall health. This study also revealed the promise of Fe-HMA as a superior iron source in piglet nutrition.

## Data Availability

The datasets presented in this study can be found in online repositories. The names of the repository/repositories and accession number(s) can be found below: https://www.ncbi.nlm.nih.gov/, PRJNA1192598.
