# Correction: Changes of metabolic parameters, antioxidant capacity, and gut microbiota in response to substitution of ferrous sulfate with iron hydroxy methionine analog chelate in weaned piglets

**DOI:** 10.3389/fcimb.2026.1902941

**Published:** 2026-06-24

**Authors:** Yuemeng Fu, Guohui Zhou, Yuhang Liu, Xuejun Yuan, Ning Jiao, Wenbiao Lu, Weiren Yang

**Affiliations:** 1 Key Laboratory of Efficient Utilization of Non-Grain Feed Resources (Co-construction by Ministry and Province), Ministry of Agriculture and Rural Affairs, Shandong Provincial Key Laboratory of Animal Nutrition and Efficient Feeding, College of Animal Science and Technology, Shandong Agricultural University, Tai’an, China; 2 College of Life Sciences, Shandong Agricultural University, Tai’an, China; 3 Research and Development Department, Fujian Syno Biotech Co., Ltd., Fuzhou, China

**Keywords:** antioxidant, gut microbiota, metabolic parameters, organic iron, weaned piglets

A correction has been made to the section 2 **Material and methods**, 2.2 *Piglets and management*:

In the published article, there was an error in the company name for the material provider. The company was displayed as “Fujian Syno Biotech Co., Ltd.”. The correct company name is “Changsha Xinjia Bio-Engineeriong Co., Ltd.’’

The original version of this article has been updated.

